# Hole Transport Layer-Free CsSnI**_3_**‑Based Perovskite Solar Cells with WS**__2__** Electron Transport Layer: Design, Simulation, and Performance Optimization

**DOI:** 10.1021/acsomega.6c00278

**Published:** 2026-05-15

**Authors:** Rukon Uddin, Hidir Duzkaya

**Affiliations:** † Graduate School of Applied and Natural Sciences, 37511 Gazi University, Ankara 06560, Turkey; ‡ Department of Electrical-Electronic Engineering, Gazi University, Ankara 06560, Turkey

## Abstract

Perovskite solar cells (PSCs) have garnered significant attention due to their outstanding optoelectronic properties and ease of fabrication. However, their commercialization is hindered by high costs and stability issues associated with the Hole Transport Layer (HTL) and the high cost of back contacts. In this study, a novel, cost-effective, HTL-free PSC structure using a lead-free CsSnI_3_ absorber and WS_2_ electron transport layer (ETL) is designed and optimized using SCAPS-1D simulation. The device performance was enhanced by systematically optimizing key parameters, including layer thickness, doping concentration, defect density, and series/shunt resistance. The optimized cell, utilizing a Nickel (Ni) back contact, achieved a Power Conversion Efficiency (PCE) of 26.08%, with an open-circuit voltage (V_oc_) of 0.94 V, a short-circuit current (J_sc_) of 34.84 mA/cm^2^ and a fill factor (FF) of 79.89%. This design offers a highly efficient, nontoxic, and economically viable alternative by replacing traditional, expensive HTL/metal contact combinations (>500 euros/g) with Ni, which costs only ∼2.23 euros/g.

## Graphical abstract

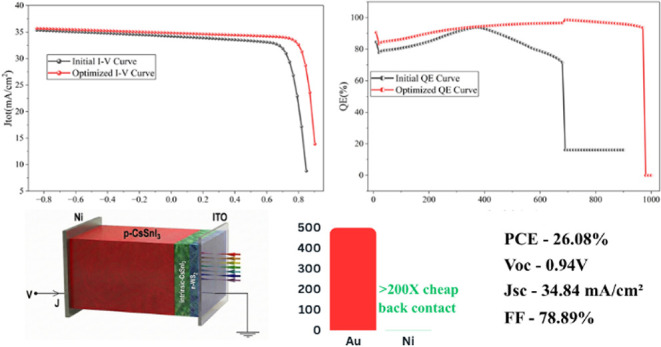

## 1 Introduction

Perovskite materials, named after the mineral calcium titanate (CaTiO_3_), have emerged as a cornerstone in advanced materials science due to their remarkable structural versatility and exceptional physical properties. Characterized by the general formula ABX_3_, perovskites are known for their unique crystallographic structure, where a large cation (A) occupies the cuboctahedral voids, a smaller cation (B) resides at the octahedral centers, and an anion (X) links the network. This structural framework enables a wide range of compositional tuning, resulting in diverse optoelectronic properties (1,2). Initially investigated for ferroelectric and superconducting applications, the discovery of their exceptional semiconducting properties revolutionized their role in photovoltaics.^3^

Perovskites exhibit several intrinsic characteristics that make them suitable for optoelectronic applications, particularly in solar cells. These include the optimal bandgap of perovskite materials; many perovskite materials exhibit bandgaps between 1.1 and 1.7 eV, which are ideal for sunlight absorption.^4,5^ Perovskite materials also have high absorption coefficients. Hence, they require thinner layers to absorb sunlight effectively.^6^ Perovskites facilitate efficient charge transport due to high electron and hole mobilities.^7,8^ Unlike silicon, perovskites are relatively tolerant to defects, allowing for efficient carrier dynamics even in imperfect crystals.^9^ Their solution-processable nature enables inexpensive production methods, including spin-coating and inkjet printing.^10,11^

The advent of perovskite solar cells (PSCs) has disrupted the photovoltaic landscape, demonstrating an unprecedented improvement in power conversion efficiency (PCE) from 3.8% in 2009 to over 25% by 2024.^1,12^ Their exceptional efficiency, combined with low fabrication costs and the potential for lightweight, flexible devices, positions PSCs as a transformative technology for renewable energy.^2^ Despite these advantages, challenges such as stability, the toxicity of lead-based compositions, and the high material costs of traditional hole transport layers (HTLs) remain obstacles to commercialization.^13,14^

Cesium tin iodide (CsSnI_3_) has emerged as a promising lead-free perovskite material, addressing environmental and health concerns associated with lead-based perovskites.^2,15^ CsSnI_3_ features a direct bandgap of approximately 1.3 eV, high absorption coefficients (>10^4^ cm^–1^), and excellent carrier mobilities (up to 300 cm^2^/(V.s)). These properties make CsSnI_3_ suitable for photovoltaic applications, providing an eco-friendly alternative without compromising efficiency.^16,17^

However, CsSnI_3_ faces intrinsic challenges. Oxidation of Sn^2+^ to Sn^4+^ leads to self-doping and degradation, reducing the material’s effectiveness.^18^ CsSnI_3_ rapidly degrades under ambient conditions, necessitating encapsulation strategies or intrinsic stabilization^9,19^ and high defect densities can lead to charge recombination and reduced photovoltaic performance.^20^

Incorporating CsSnI_3_ into PSCs has yielded promising results. Various device architectures, such as planar and mesoporous structures, have been explored to harness its properties.^11^ Enhancements in encapsulation techniques and the incorporation of stabilizing additives like SnF_2_ or tin halides have mitigated some stability issues.^2^ Recent studies demonstrate CsSnI_3_-based PSCs achieving PCEs exceeding 20%, showcasing their potential in next-generation photovoltaics.^9,17^

Despite significant advancements, key research gaps persist in the CsSnI_3_-based PSCs. Conventional designs rely on expensive and unstable HTLs, such as Spiro-OMeTAD, which increase costs and compromise stability.^9,11^ Suboptimal alignment between the CsSnI_3_ absorber and charge transport layers leads to recombination losses.^17,20^ The use of costly materials, like gold in back contacts, further escalates device costs.^14^ A systematic approach to optimizing device parameters, including doping density, defect states, and layer thickness, is often lacking.^15,21^

This study proposes a novel HTL-free CsSnI_3_-based PSC structure, incorporating tungsten disulfide (WS_2_) as the electron transport layer (ETL) and nickel (Ni) as the back-contact. WS_2_ offers high electron mobility, chemical stability, and effective energy level alignment with CsSnI_3_, addressing interface engineering challenges.^17,22^ The elimination of HTL and the use of cost-effective Ni as the back contact address cost and stability concerns, respectively.^15,23^

The proposed structure not only simplifies fabrication but also enhances the economic viability and long-term stability of the CsSnI_3_-based PSCs. By systematically optimizing the absorber layer thickness, doping concentration, and interface properties, this study aims to achieve high efficiency while minimizing recombination losses. The insights gained are expected to contribute to the scalability and commercialization of lead-free PSCs.^9,24^

This research explores the synergy between material engineering and device architecture to overcome longstanding challenges in PSCs. By leveraging the unique properties of CsSnI_3_ and WS_2_ and through meticulous simulation and optimization, the study underscores the potential of sustainable and efficient photovoltaics.^17,20^

## 2 Modeling and Simulation

SCAPS-1D, developed by the ELIS Department of Ghent University, is a widely used numerical simulation platform for investigating the performance of perovskite solar cells (PSCs). On the basis of well-established semiconductor device physics, SCAPS-1D^21,24,25^ enables detailed modeling of diverse device configurations and material stacks. The software comprehensively simulates the fundamental photovoltaic processes, including optical absorption, photogeneration of charge carriers, carrier transport and extraction, and recombination mechanisms within the device.

The electrostatic behavior of the device is governed by Poisson’s equation,^26^ which relates the spatial variation of the electric potential to the local charge distribution. This relationship is expressed as:

1 $$\frac{d^{2}\psi \left(x\right)}{dx^{2}}=\frac{q}{\varepsilon_{0}\varepsilon_{r}}\left[p\left(x\right)-n\left(x\right)+N_{D}-N_{A}+\rho_{p}-\rho_{n}\right]$$

Where ψ represents the electrostatic potential, ε_r_ is the relative dielectric constant of the material, and ε_0_ denotes the vacuum permittivity. N_D_ and N_A_ correspond to donor and acceptor doping concentrations, respectively, while n and p indicate the free electron and hole densities. The quantities ρ_n_ and ρ_p_ refer to the charge densities of electrons and holes, and q is the elementary charge.

Charge conservation for electrons and holes is described by the continuity equations:

2 $$\frac{\partial n}{\partial t}=\frac{1}{q}\frac{\partial J_{n}}{\partial x}+\left(G_{n}-R_{n}\right)$$

3 $$\frac{\partial p}{\partial t}=\frac{1}{q}\frac{\partial J_{p}}{\partial x}+\left(G_{p}-R_{p}\right)$$

In these expressions, J_n_ and J_p_ denote the electron and hole current densities, respectively. The terms G_n_ and G_p_ represent the generation rates of electrons and holes, whereas R_n_ and R_p_ account for the respective recombination rates.

Carrier transport in PSCs is modeled using the drift–diffusion formalism, which defines the current densities of electrons and holes as:

4 $$J_{n}=q\mu n_{n}n\varepsilon +qD_{n}\partial n$$

5 $$J_{p}=q\mu n_{p}p\varepsilon +qD_{p}\partial p$$

Here, D_n_ and D_p_ are the diffusion coefficients for electrons and holes, respectively, and μ_n_ and μ_p_ denote their mobilities.

SCAPS-1D numerically solves Poisson’s equation together with the carrier continuity equations across the device layers, including interfaces and electrical contacts. The fill factor (FF), which reflects the quality of the current–voltage characteristics of the solar cell, is calculated using^27^

6 $$FF=\frac{\left(J_{mp}\times V_{mp}\right)}{\left(J_{sc}\times V_{oc}\right)}$$

where V_mp_ and J_mp_ correspond to the voltage and current density at the maximum power point, respectively. J_sc_ represents the short-circuit current density, and V_oc_ is the open-circuit voltage.

Finally, the power conversion efficiency of the solar cell is determined by normalizing the maximum output power to the incident optical power P_in_, which is defined under standard AM 1.5G solar illumination conditions:^28^

7 $$\eta =\frac{\left(V_{oc}\times J_{sc}\times FF\right)}{P_{in}}$$

Figure 1 indicates the proposed structure of the study. The structure consists of a combination of front and back contacts, WS_2_ as the ETL, and CsSnI_3_ as the absorber layer. The parameters used in the study are listed in Table 1.

**Figure 1:**
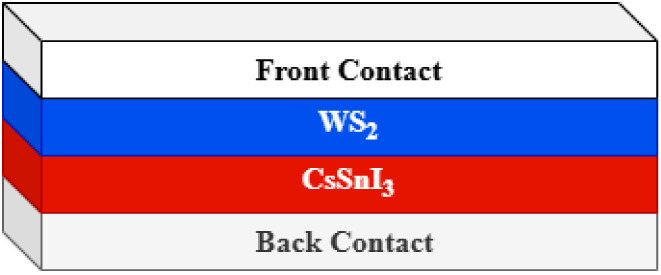
Proposed cell structure.

**Table 1:** Parameters of the Absorber Layer and ETL

Parameters (unit)	CsSnI_3_ ^29^	WS_2_ ^15^
Thickness (μm)	0.5	0.08
Bandgap (eV)	1.27	1.80
Electron affinity (eV)	4.47	3.95
Dielectric Permittivity	18.00	13.60
Density of states N_c_ (cm^–3^)	1.8 × 10^19^	1.8 × 10^19^
Density of states N_v_ (cm^–3^)	2.2 × 10^18^	2.2 × 10^18^
Electron thermal velocity (cm/s)	1 × 10^7^	1 × 10^7^
Hole thermal velocity (cm/s)	1 × 10^7^	1 × 10^7^
Electron mobility (cm^2^/(V s))	4.37	100
Hole mobility (cm^2^/(V s))	4.37	100
Donor doping density (cm^–3^)	0	1 × 10^18^
Acceptor doping density (cm^– 3^)	1 × 10^17^	0
Defect density (cm^–3^)	1 × 10^14^	1 × 10^18^

Figure 2 provides the band alignment of the proposed cells. The diagram clearly shows that the cell has an organized band alignment for electron–hole pair collection, and hence, it can be useful for designing an efficient cell.

**Figure 2:**
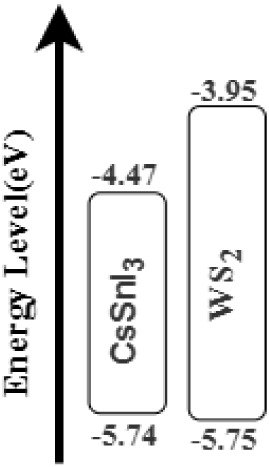
Band alignment.

## 3 Results and Discussion

The initial simulation of the proposed HTL-free CsSnI_3_-based perovskite solar cell with WS_2_ ETL, without any parameter optimization, yielded a power conversion efficiency (PCE) of 22.38%, while the conventional ETL TiO_2_ can provide a PCE of only 10.61%, and SnO_2_ can provide a PCE of 16.19% for a similar HTL-free structure. The quantum efficiency analysis in Figure 3 shows a continuous drop in efficiency after a certain wavelength, indicating suboptimal light absorption and charge-carrier extraction. This initial performance, while promising, highlights the need for further optimization to enhance efficiency and stability. Similar trends have been reported in previous studies, where initial efficiency gains were hindered by suboptimal carrier dynamics and material defects.^1,11^

**Figure 3:**
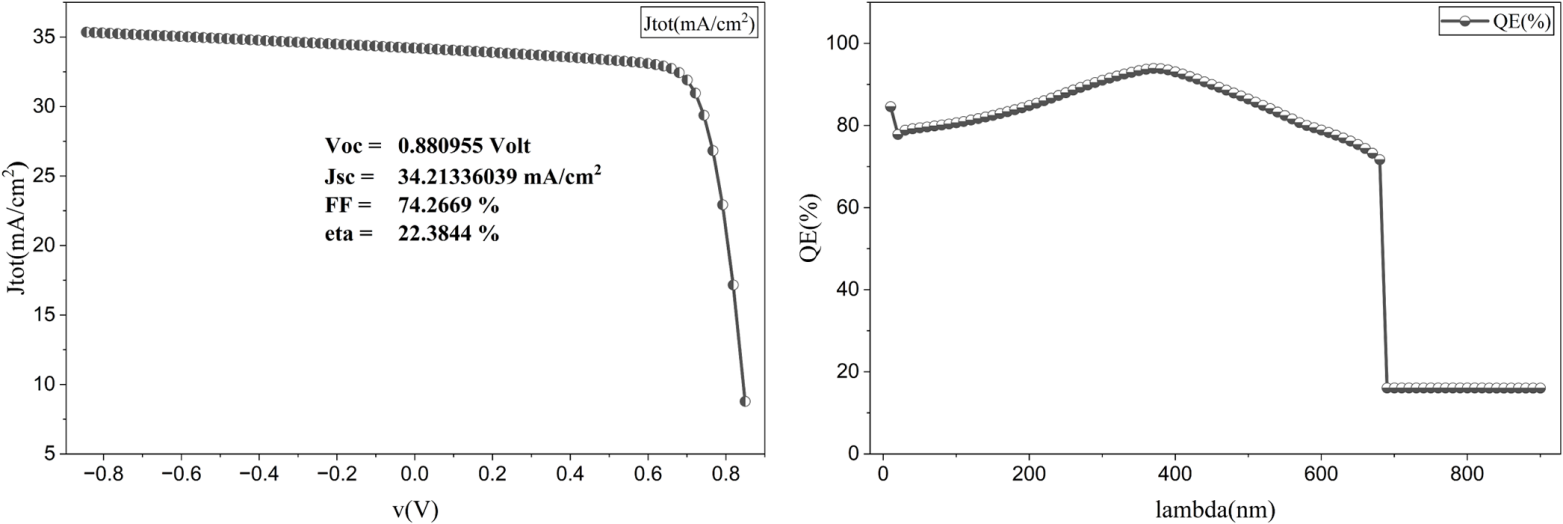
Initial J–V curve of the proposed cell and the quantum efficiency curve of the proposed cell.

In earlier works, Kojima et al.^1^ identified similar issues in the initial stages of perovskite solar cell development, citing recombination losses as a primary factor limiting the efficiency. Additionally, Fatima et al.^11^ demonstrated that achieving optimal efficiency requires careful fine-tuning of layer properties to address inherent losses due to nonuniform carrier distribution.

Hence, the cell is not optimized, and it is possible to optimize the cell’s parameters, allowing for higher PCE and quantum efficiency to be achieved. Hence, the cell is simulated by varying parameters to optimize its performance.

### 3.1 Impact of Thickness

The thicknesses of the absorber and electron transport layer (ETL) play a crucial role in determining the overall performance of perovskite solar cells (PSCs), influencing parameters such as light absorption, charge extraction, resistive losses, and recombination rates. Optimizing these layers is essential for maximizing the power conversion efficiency (PCE) and device stability.

The simulation results in Figure 4 reveal a nearly linear improvement in cell performance with increasing absorber layer thickness. Specifically, the open-circuit voltage (V_oc_), short-circuit current density (J_sc_), and overall efficiency increase as the thickness increases, whereas the fill factor (FF) initially declines before improving slightly. The optimal absorber thickness of 1 μm yields a PCE of 23.43%, a V_oc_ of 0.897 V, a J_sc_ of 34.56 mA/cm^2^, and an FF of 75.59%. Similar trends have been reported in previous studies, where an absorber thickness of around 1 μm has been found to provide an optimal balance between light absorption and charge collection efficiency.^1,11^

**Figure 4:**
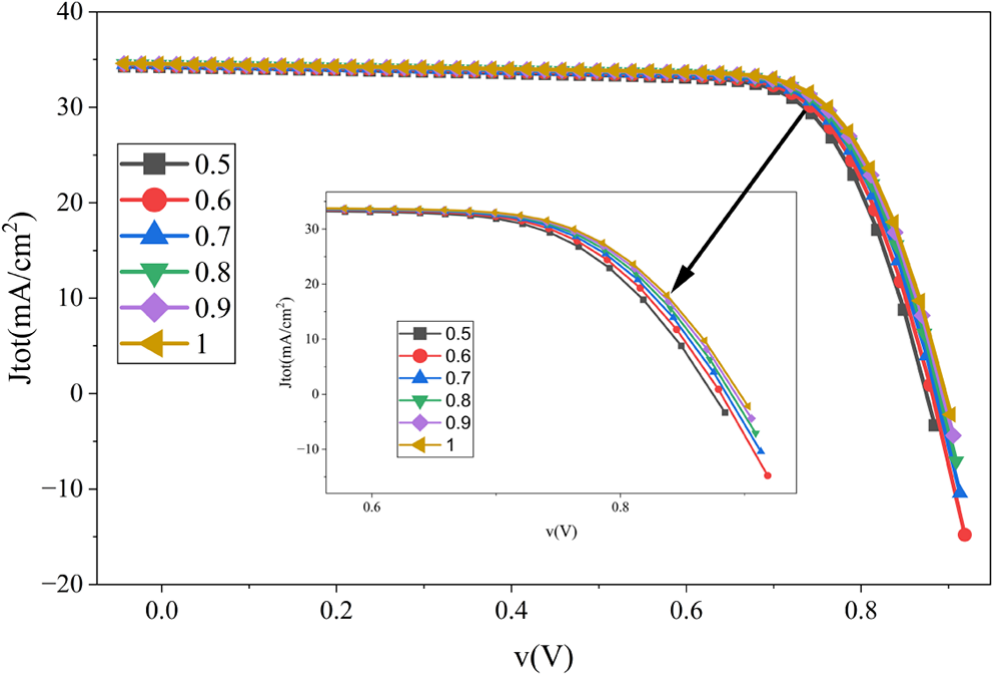
Influence of absorber layer thickness on the JxV curve of the proposed cell.

The reason for the improved output is illustrated in Figure 5. It is evident from Figure 5 that increasing the absorber thickness can move the intensity of the electric field as well. Here, the electric field drops sharply from around 50000 V/cm to −380000 V/cm at the edge of the absorber/ETL and then increases sharply. The locus of the sharp electric field changes linearly with the thickness of the absorber. For the case of cumulative generation, the maximum generation rate is around 35 mA/cm^2^, and it drops sharply at the final section of the absorber material, while the drop is less sharp for the case of the ETL. It can also be found that a longer absorber thickness can improve the overall generation of the proposed cell.

**Figure 5:**
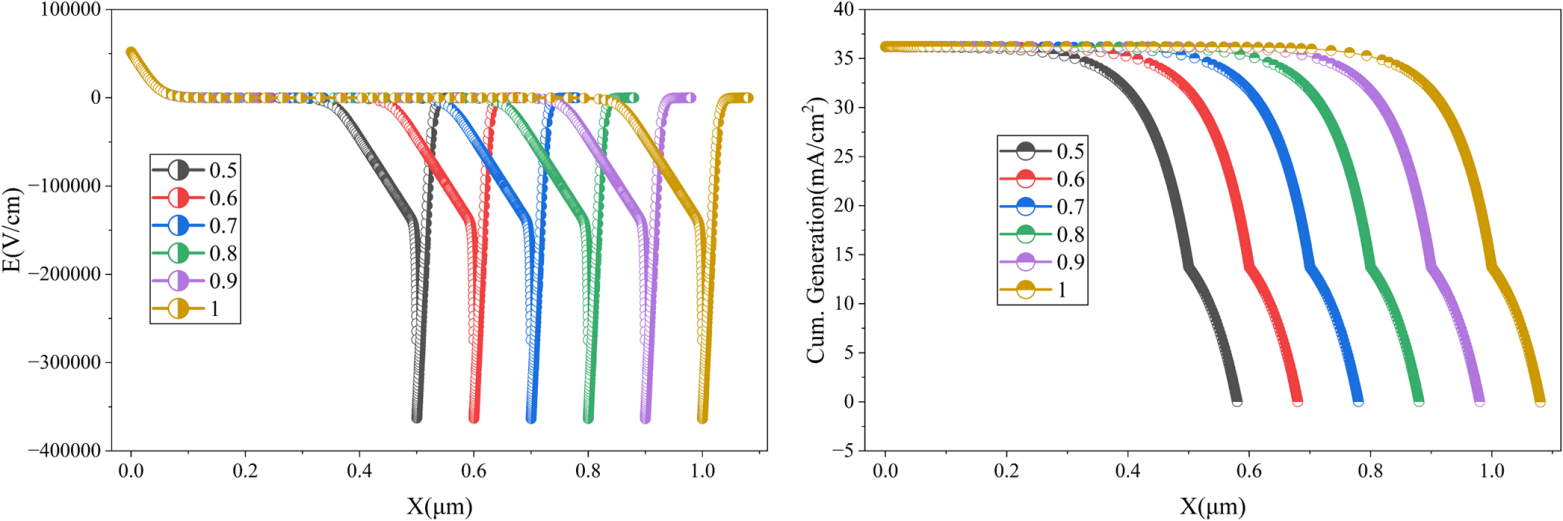
Impact of absorber layer thickness on the electric field distribution and cumulative generation.

While Figure 6 shows that the generation rate of the cell changes significantly with the thickness of the ETL. The maximum generation rate (∼3.5 × 10^22^ 1/(cm^3^.s)) can be achieved with a lower thickness (0.03 μm). However, the generation rate is comparatively lower at different parts of the cell. When the ETL thickness is 0.05 μm, the cell has a favorable generation rate (with a maximum generation rate of ∼2.9 × 10^22^ 1/(cm^3^.s)) for the cell’s overall thickness and can provide optimal output. This behavior is attributed to the interplay between carrier transport and optical interference effects. The optimal ETL thickness of 0.05 μm is selected to achieve a PCE of 23.86%, a V_oc_ of 0.896 V, a J_sc_ of 35.65 mA/cm^2^, and an FF of 74.68%. This aligns with findings from prior research, where thinner ETLs have shown higher efficiency, but slightly thicker layers are preferred for improved mechanical stability and ease of fabrication.^6,15^

**Figure 6:**
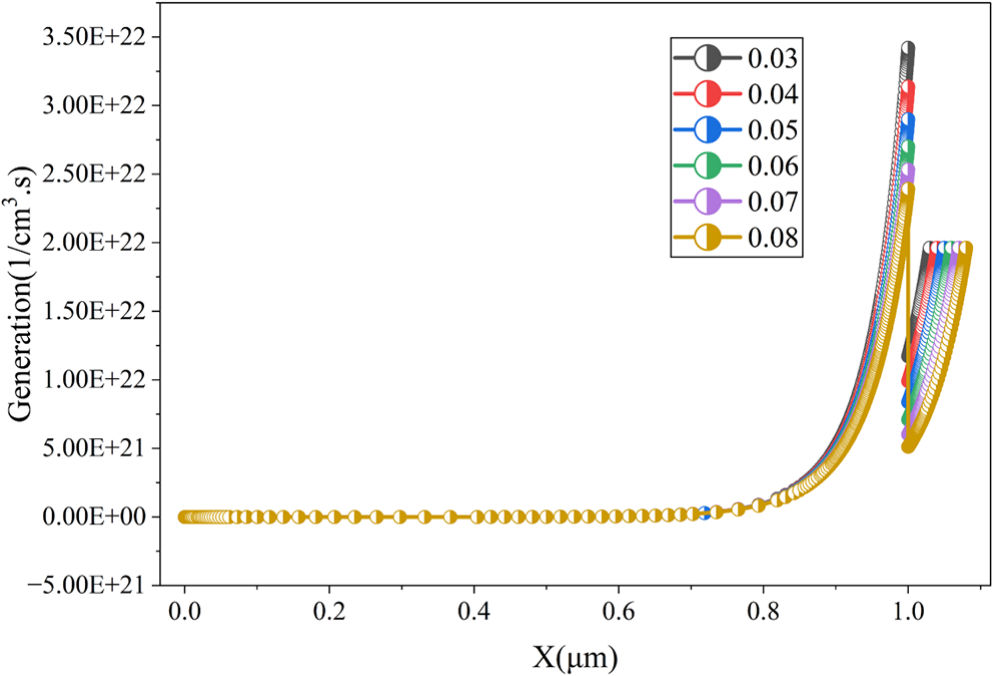
Impact of ETL thickness on the generation rate of the proposed cell.

Comparing the results with previous studies, similar outcomes have been observed in the literature. For instance, Stranks et al.^6^ reported that a 1 μm thick perovskite layer achieves an optimal balance between light absorption and charge carrier diffusion length, while Sobayel et al.^15^ emphasized that WS_2_ ETL thickness in the range of 0.07–0.1 μm provides the best compromise between charge transport and fabrication feasibility. Additionally, Lee et al.^10^ suggested that excessively thin ETLs can lead to charge accumulation and recombination losses, further supporting the choice of 0.05 μm.

Therefore, based on these findings and prior research, the selected thickness values provide an optimal trade-off between efficiency and practicality, ensuring the device’s feasibility for large-scale manufacturing while maintaining high performance.

### 3.2 Impact of Doping

Doping plays a crucial role in enhancing the efficiency of perovskite solar cells (PSCs). It increases the concentration of charge carriers, thereby improving conductivity, enhancing charge mobility, and reducing recombination losses by mitigating trap states within the absorber layer. Proper doping levels are essential for balancing carrier injection and extraction, ensuring optimal device performance and stability.

The simulation results in Figure 7 show that the cell performance is initially low at lower doping concentrations. As the absorber doping concentration increases to 5 × 10^19^ cm^–3^ with a parabolic grading to 1 × 10^16^ cm^–3^, the power conversion efficiency (PCE) reaches its peak at 27.27%. Beyond this concentration, efficiency drops sharply, indicating increased recombination due to excess carrier density and potential defect-induced losses. The influence of absorber layer doping can be described by Figure 8, where it is evident that the cell exhibits spontaneous electron–hole emission when the doping density is 5 × 10^19^ cm^–3^. Similar trends have been observed in previous studies, where optimal doping levels were found to be critical in achieving maximum efficiency without introducing significant nonradiative recombination pathways.^1,11^

**Figure 7:**
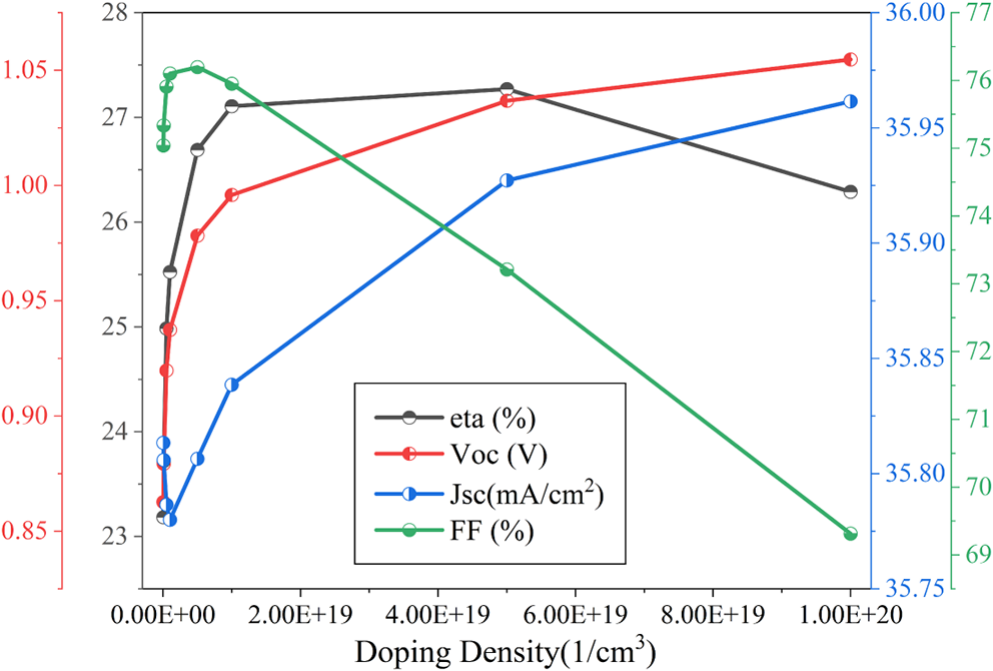
Impact of absorber layer doping on the cell performance.

**Figure 8:**
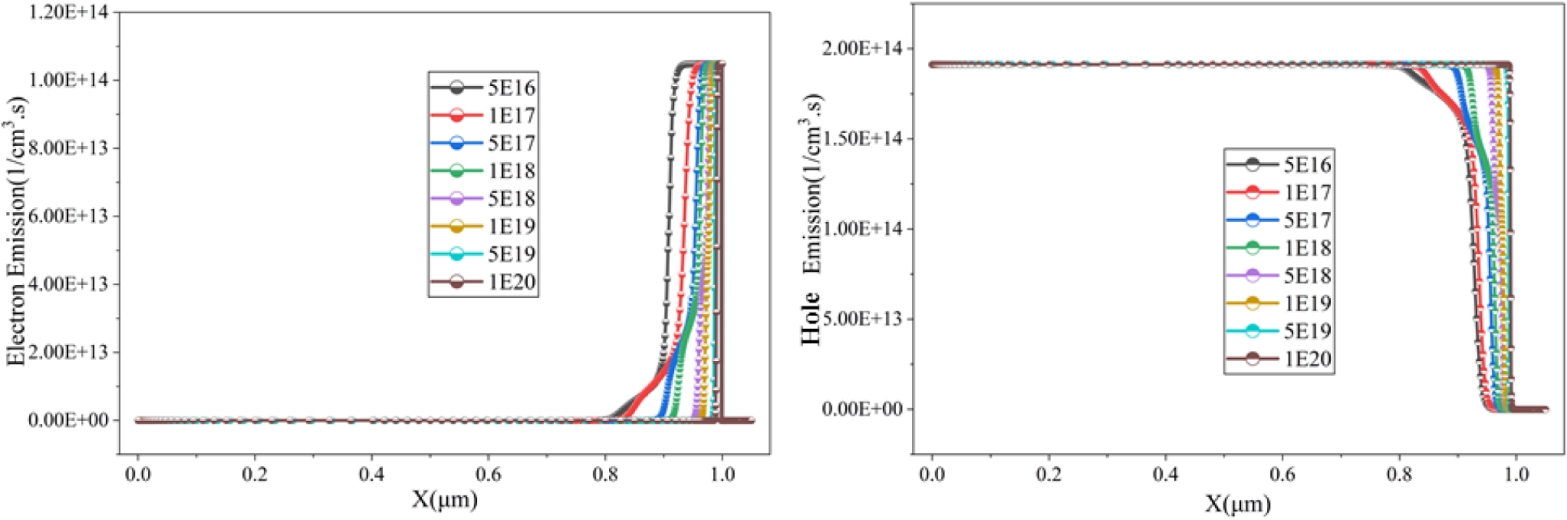
Absorber doping impact on the electron and hole emission in the proposed cell.

Since this study excludes the hole transport layer (HTL), a lower doping concentration in the absorber layer can induce an interface barrier between the absorber and the back contact, leading to energy misalignment and charge accumulation. To counteract this issue, a thick absorber layer is selected, allowing for a doping gradient that facilitates better carrier transport and reduces interfacial resistance and recombination losses. As suggested by Lee et al.,^10^ doping gradients can enhance charge collection efficiency by creating an internal electric field that drives charge carriers toward their respective contacts.

In this study, the absorber is doped with a parabolic grading, starting with a peak value of 5 × 10^19^ cm^–3^ and a minimum value of 1 × 10^16^ cm^–3^. This graded doping profile effectively reduces recombination losses and enhances charge extraction efficiency. This approach is consistent with findings by Sobayel et al.,^15^ who demonstrated that graded doping profiles in perovskite absorbers substantially improve device performance by balancing charge carrier dynamics. Furthermore, advanced doping techniques, such as codoping strategies with alkali metals or halide treatments, have been shown to further enhance stability and efficiency by passivating defects and improving crystallinity.^17,18^

Several studies have demonstrated the importance of optimized doping in perovskite solar cells. Stranks et al.^6^ reported that doping optimization leads to a significant reduction in trap-assisted recombination, while Hossain et al.^25^ emphasized the importance of graded doping in improving the built-in electric field and the carrier transport properties. The results from this study align well with these findings, showcasing the critical role of doping concentration and distribution in achieving high-efficiency, stable devices.

Whereas ETL doping density (Figure 9) has an oscillating impact on the performance of the proposed cell starting with significantly low output performance; it improved gradually but then dropped again. The reason behind this can be observed in Figure 10. It is clear from Figure 10 that the generation improves with the doping density; however, the recombination drops significantly with the doping density increment, and the most favorable generation-recombination can be found when the doping density is 5 × 10^18^ cm^–3^, providing a power conversion efficiency of 29.39%, V_oc_ of 1.03 V, 35.28 mA/cm^2^, and an FF of 80.84%.

**Figure 9:**
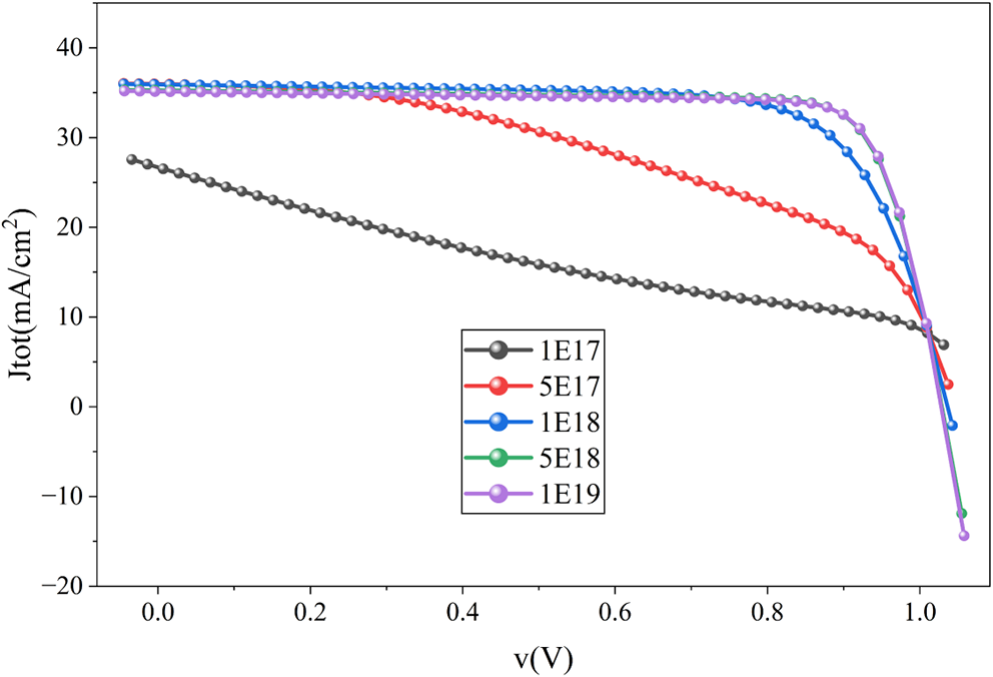
Impact of ETL doping density on the performance of the proposed cell.

**Figure 10:**
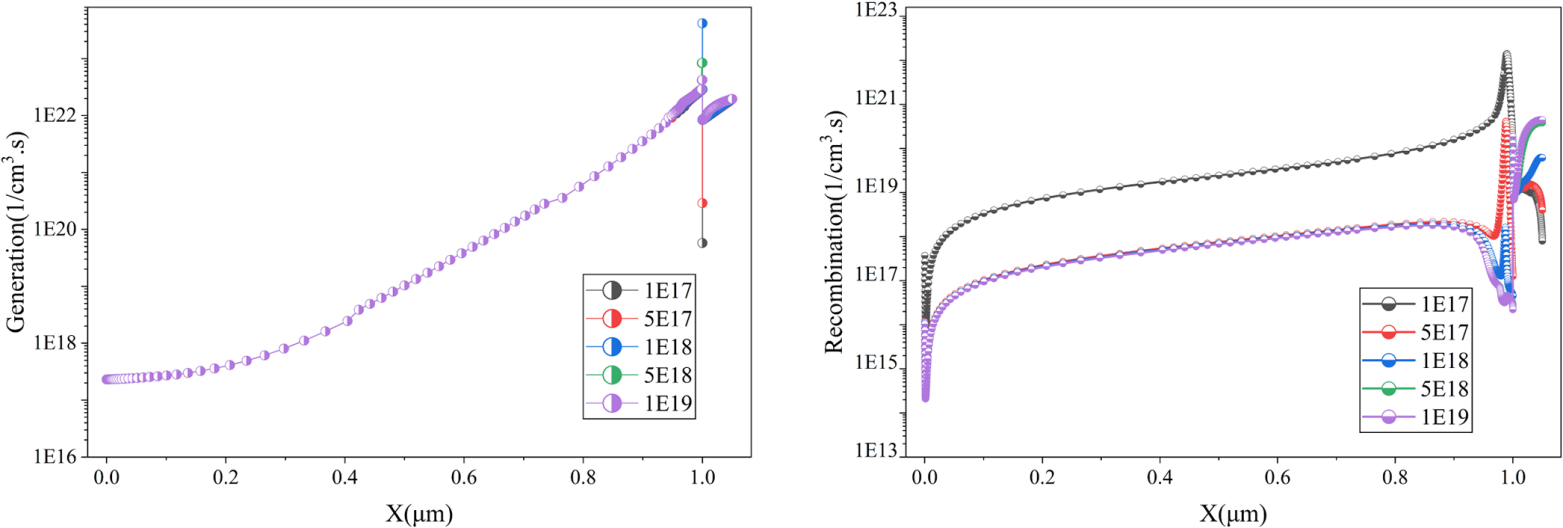
Impact of ETL doping density on the generation and recombination rate of the cell.

### 3.3 Impact of Absorber Defect Density

Defects in perovskite solar cells (PSCs) introduce trap states that reduce carrier lifetime and increase recombination rates, leading to efficiency losses. The simulation results in Figure 11 indicate that increasing the defect density severely degrades performance, with the power conversion efficiency (PCE) declining significantly at a defect density of 1 × 10^18^ cm^–3^. Figure 12 indicates that the rising defect density does not contribute to the generation of an e–h pair; however, it is a major reason for the sharp increase in recombination. It is observed that recombination is lowest at 1 × 10^14^ cm^–3^, while it peaks at 1 × 10^19^ cm^–3^. To minimize recombination losses. The ideal defect density for CsSnI3 based material is ∼1 × 10^14^ cm^–3^.^30,31^ An optimized defect density of 1 × 10^14^ cm^–3^ is selected for the current study, resulting in a PCE of 29.39%. Similar optimization strategies have been reported in previous studies, emphasizing the importance of low defect densities for enhanced charge carrier dynamics and performance^1,11^

**Figure 11:**
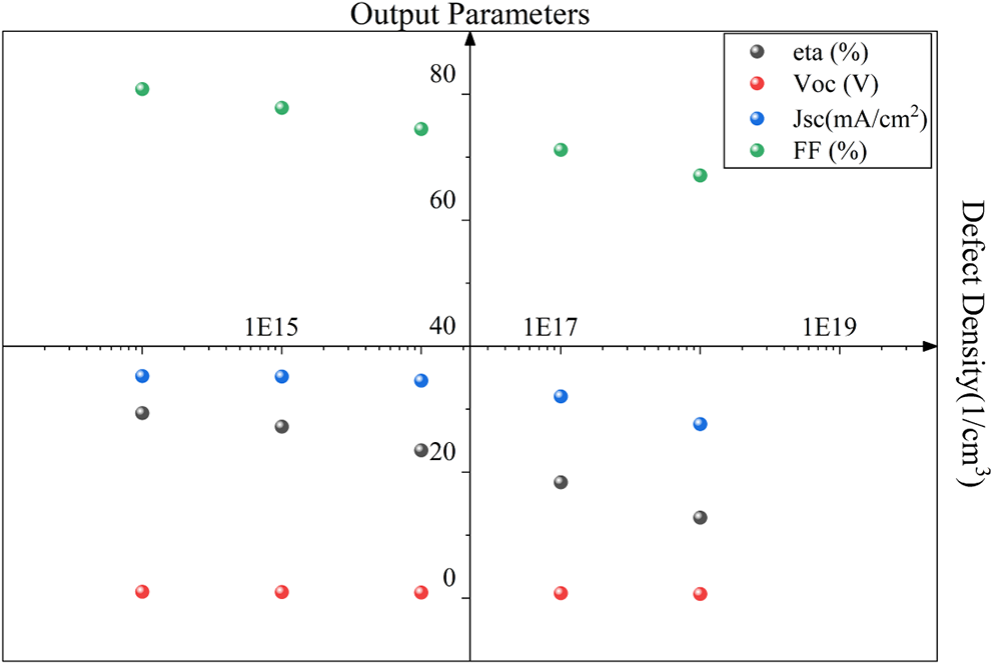
Impact of absorber defect density on the cell performance .

**Figure 12:**
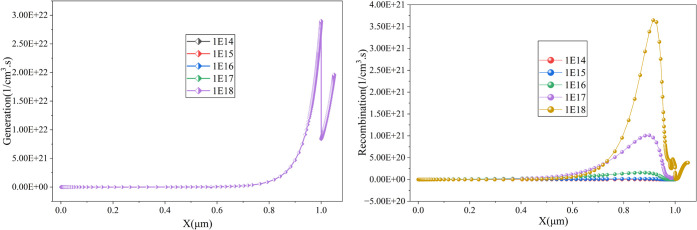
Impact of absorber defect density on the generation-recombination profile of the cell.

Simulation-based studies, such as those by Hossain et al.^25^ and Lin et al.^17^ confirm that defect passivation techniques, including optimized fabrication processes and material engineering, are crucial in reducing trap states and enhancing efficiency. The results from this study align with these findings, highlighting the necessity of minimizing defects to achieve superior photovoltaic performance.

Previous studies have demonstrated that maintaining defect densities below 1 × 10^15^ cm^–3^ significantly enhances V_oc_ and FF by reducing nonradiative recombination.^11^ The study’s findings corroborate these results, demonstrating substantial efficiency improvements with optimized defect levels, thereby making the proposed PSC design more viable for practical applications.

### 3.4 Impact of Recombination and Interface

Previously, any recombination or interface between layers was not considered. In this part of the study, recombination and the interface between the absorber and ETL are considered. The radiative recombination coefficient is chosen as 3 × 10^–11^ cm^3^/s, and the Auger electron and hole capture coefficient as 1 × 10^–29^ cm^6^/s.^32^ For the defect of the CsSnI_3_/WS_2_ interface, the capture cross-sections for electrons and holes are considered to be 1 × 10^–19^ cm^2^, the total density (integrated over all energies) is 1 × 10^12^ 1/cm^2^, and the density at the peak energy is 5.64 × 10^12^ 1/cm^2^ eV. While the energy distribution is the Gaussian type, the energy with respect to the reference (eV) and the characteristic energy (eV) are, respectively, 0.6 and 0.1. After optimizing these parameters, the PCE of the cell dropped to 24.66%.

Figure 13 illustrates the decline in output performance resulting from the introduction of recombination and the interface layer in the cell. Figure 14 illustrates the reduction in quantum efficiency resulting from this adjustment. The optimized cell exhibits a power conversion efficiency of 24.66%, with a V_oc_ of 0.94 V, J_sc_ of 32.60 mA/cm^2^, and FF of 80.12%, respectively.

**Figure 13:**
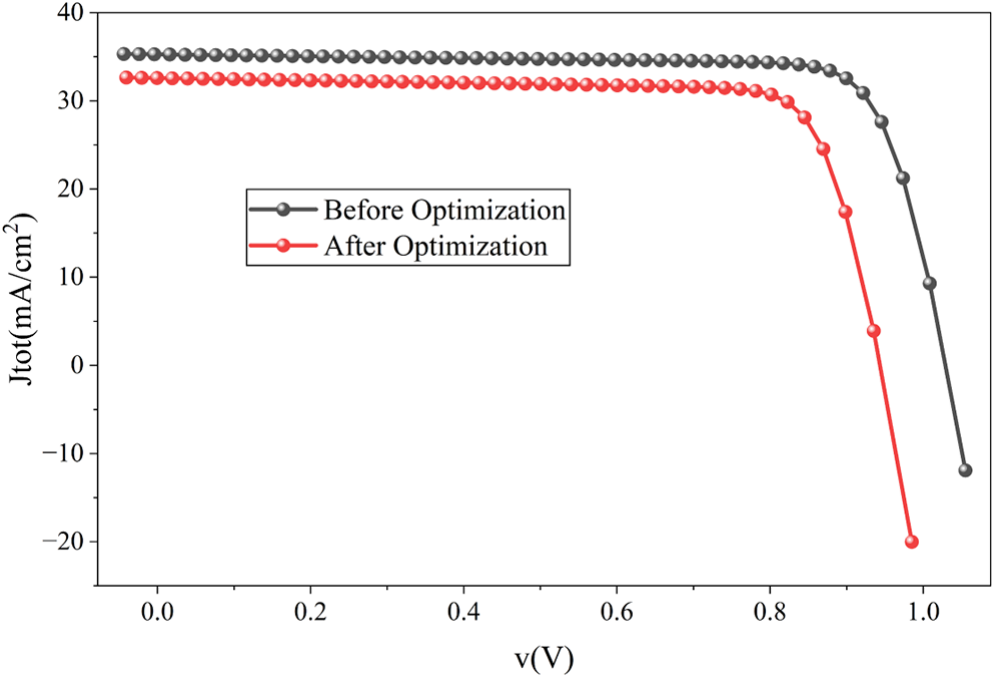
J-V curve comparison before and after optimization of recombination and interface layer defect density of the proposed cell.

**Figure 14:**
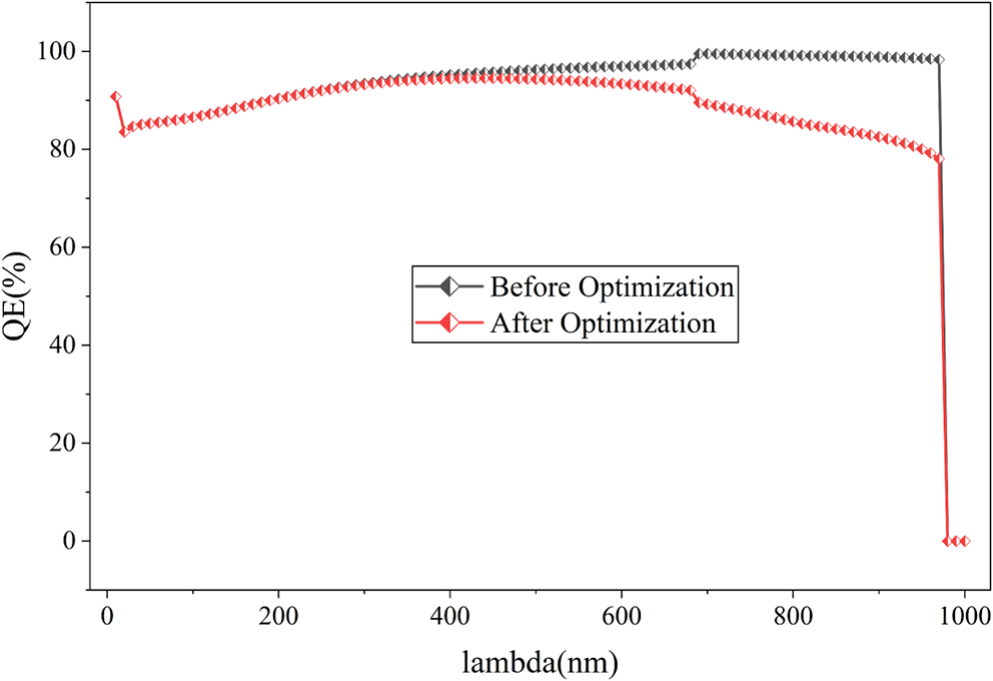
Quantum efficiency curve comparison before and after optimization of recombination and interface layer defect density of the proposed cell.

### 3.5 Impact of Resistance

Series and shunt resistances are crucial factors that significantly influence the overall performance of perovskite solar cells (PSCs). The simulation results in Figure 15 demonstrate a linear relationship between series resistance and fill factor (FF), with FF being the most affected parameter, while power conversion efficiency (PCE) is slightly influenced, and short-circuit current density (J_sc_) and open-circuit voltage (V_oc_) show minimal variation. An optimal series resistance of 1.25 Ω is chosen, resulting in a PCE of 24.66%, V_oc_ of 0.94 V, J_sc_ of 32.60 mA/cm^2^, and FF of 80.12%.

**Figure 15:**
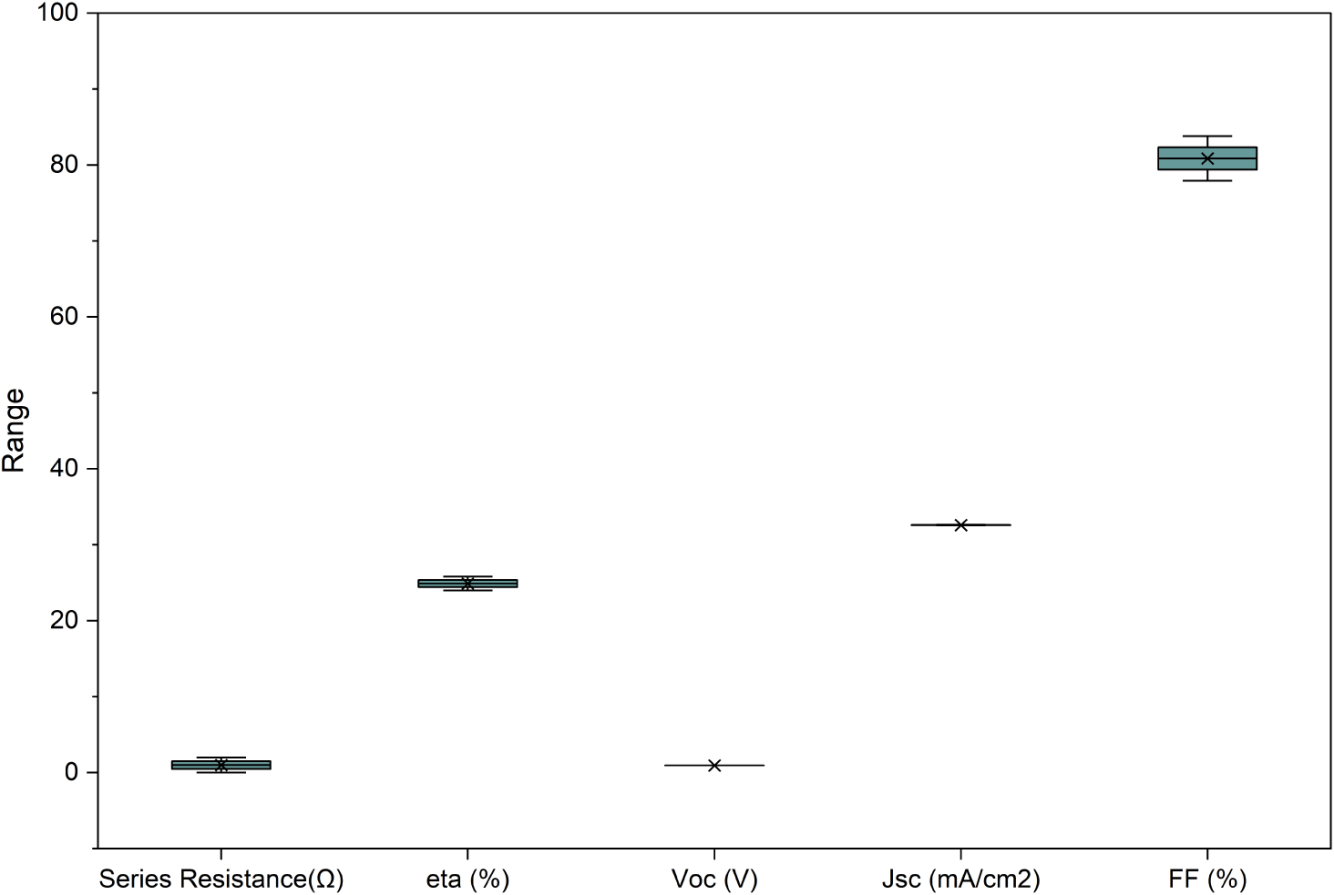
Impact of series resistance on the performance of the cell.

The addition of a 1000 Ω shunt resistance maintained an efficiency of 24.66%, highlighting the significant impact of leakage paths on device performance. These findings align with previous studies that emphasize the importance of optimizing series and shunt resistances to minimize power losses and enhance charge transport efficiency.^1,11^

Studies by Hossain et al.^25^ have shown that higher series resistance leads to voltage drops across the device, affecting the fill factor and reducing efficiency. Similarly, Lin et al.^33^ reported that optimizing the shunt resistance helps to suppress leakage currents, thereby improving operational stability.

Prior research suggests that minimizing series resistance to values below 2 Ω can lead to improved fill factors, while maintaining shunt resistance at high values helps achieve higher efficiencies by mitigating parasitic losses.^8,34^ Results from this study are consistent with these findings, demonstrating that effective resistance management is crucial for maximizing device performance. The simulation results confirm that series resistance has a significant influence on the fill factor, whereas shunt resistance primarily affects leakage currents and efficiency.

### 3.6 Impact of Back Contact

The back-contact is another vital element of perovskite-based solar cells. A matched back-contact is required for proper charge extraction, reducing interface recombination, and enhancing efficiency. In this study, the cell was simulated with the following back-contacts.

In this study, 10 back-contact materials were chosen, ranging the metal work function from 4.65 to 5.9 eV. The cell was then simulated by varying the back contacts. After simulation of the cell with the back contacts given in Table 2, the outcome of Figure 16 was achieved.

**Table 2:** Different Back Contact and Their Metal Work Function

Back Contact Metal^20^	Cu	Ag	Fe	C	Au	W	Ni	Pd	Pt	Se
Work Function (eV)	4.65	4.74	4.81	5	5.1	5.22	5.5	5.6	5.7	5.9

**Figure 16:**
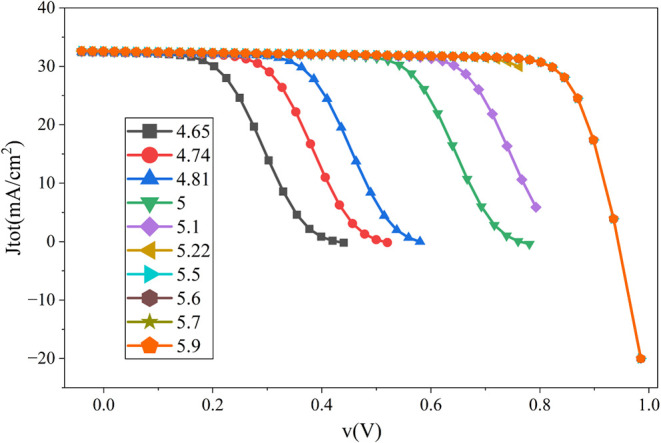
Influence of metal work function on the performance of the proposed cell.

It is clear from the graph that the cell can achieve maximum efficiency with Ni, Pt, Pd, and Se back contacts. As these contacts provide similar PCEs, the contact for the study is chosen by comparing the expenses of the contacts through Table 3. As Ni back-contact material is cheaper than other probable options, Ni is finalized as the back contact for this study. Nickel (Ni) serves as a stable back contact in perovskite solar cells due to its chemical inertness and resistance to halide-induced corrosion, which reduces the formation of metal halides at the interface and enhances device longevity. Unlike reactive metals such as Cu or Ag, Ni exhibits minimal diffusion into the perovskite layer, mitigating interfacial degradation and preserving the charge extraction efficiency. However, residual mobile ions within the perovskite, such as halide vacancies, can still migrate toward the Ni contact, potentially inducing localized recombination or minor interfacial reactions. Thermal stress during processing or operation may exacerbate these effects, but proper interface engineering, including buffer or passivation layers, can effectively suppress ion migration and stabilize the Ni/perovskite interface, ensuring both efficient charge transport and long-term operational stability.^35,36^

**Table 3:** Back Contact Materials That Provide Maximum Efficiency and Their Price

Metal Name	Metal Work Function (eV)	Price(Euro/g)^a^
Ni	5.5	2.232
Pd	5.6	635.714
Pt	5.7	230
Se	5.9	11.32

a The price of materials is taken from Sigma-Aldrich.^37^

### 3.7 Impact of Intrinsic Layer

An intrinsic layer is introduced between the CsSnI_3_ and WS_2_ layers, as shown in Figure 17, to further improve the output performance of the cells. This can reduce recombination and further enhance the power conversion efficiency of the proposed cells.

**Figure 17:**
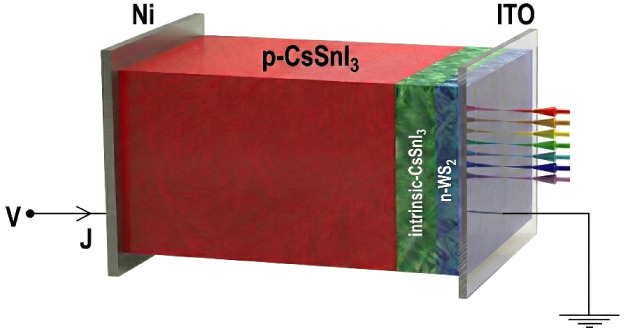
Final cell structure after the introduction of the intrinsic layer.

The cell is then simulated by varying the thickness of the CsSnI_3_ intrinsic layer from 0.05 to 0.15 μm. Introducing an intrinsic layer between the absorber and transport layers is a physically feasible and established strategy in photovoltaic device design because such interlayers are known to suppress nonradiative recombination at the absorber/transport interface and improve carrier extraction, thereby enhancing current and overall efficiency. In perovskite solar cells, engineering interfacial layers facilitates better band alignment and reduces charge recombination losses at the absorber/ETL boundary, which directly improves device performance. This approach mirrors widely used heterojunction designs, such as HIT solar cells with intrinsic passivation layers, that show higher efficiencies by reducing interface recombination and improving charge collection.^38^ According to Figure 18, although the open-circuit voltage and fill factor are dropping gradually, the cell performance and short-circuit current are increasing with thickness. The efficiency peaked at 0.12 μm and then dropped again. At 0.12 μm thickness, the optimized PCE reached 26.08%.

**Figure 18:**
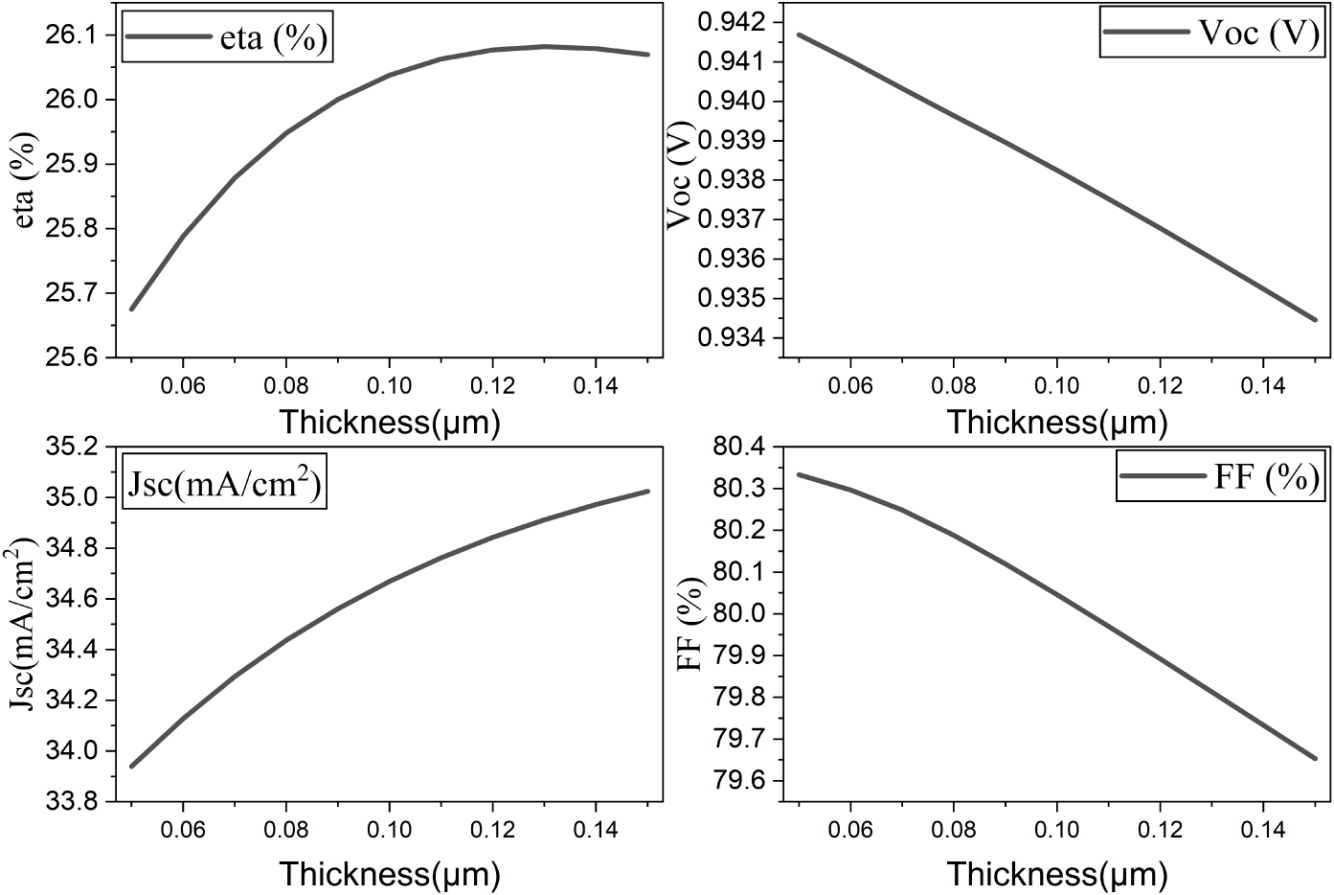
Influence of intrinsic layer thickness on the performance of the proposed cell.

The optimized cell can provide an optimal PCE of 26.08%, a V_oc_ of 0.94 V, a J_sc_ of 34.84 mA/cm^2^, and an FF of 79.89%. These output performances obey the Shockley–Queisser limit for single-junction solar cells; according to the tabulated values of the Shockley–Queisser limits, a solar cell can exhibit a maximum PCE of 32.74% when the bandgap of the absorber is 1.2 eV, and it can exhibit a maximum PCE of 32.57% when the bandgap is 1.3 eV.^19^ As CsSnI_3_ has a bandgap of 1.27 eV, it exhibits a valid output performance in accordance with the Shockley–Queisser limit.^39^

### 3.8 Impact of Temperature

Temperature has a significant influence on the performance of solar cells, affecting the charge recombination and transport mechanisms. In this section, the behavior of the proposed cell is analyzed by varying the temperature, as shown in Figure 19. The results indicate that the power conversion efficiency (PCE) is notably low at lower temperatures, primarily because of reduced carrier mobility and increased recombination rates. As the temperature increases, the PCE improves, reaching its maximum efficiency at an optimal operating temperature of 300 K. This trend is consistent with findings that moderate temperature increases enhance carrier mobility and reduce recombination barriers, thereby improving efficiency.^6,40^ Beyond 300 K, the PCE declines due to enhanced thermal excitation of charge carriers, which increases nonradiative recombination and reduces charge transport efficiency.^41^ These findings underscore the importance of maintaining optimal operating conditions to achieve maximum solar cell performance.

**Figure 19:**
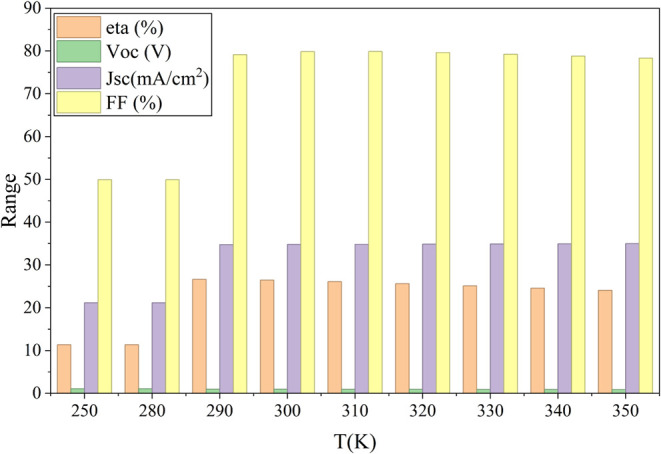
Influence of temperature on cell performance.

CsSnI_3_-based devices have shown promising progress in real ambient stability, as recent studies demonstrate that, through effective strategies such as additive engineering, surface passivation, and encapsulation, the oxidation of Sn^2+^ can be significantly suppressed. As a result, these devices are capable of maintaining stable performance for extended periods under ambient conditions, indicating strong potential for further improvement toward practical outdoor applications.^42^

### 3.9 Performance Improvement Analysis

The J–V curve and the quantum efficiency curve in Figure 20 clearly identify the improvement of the cell before and after optimization. The quantum efficiency of the cells has improved significantly, which is why the cell provides optimal efficiency, even after considering recombination and resistive losses.

**Figure 20:**
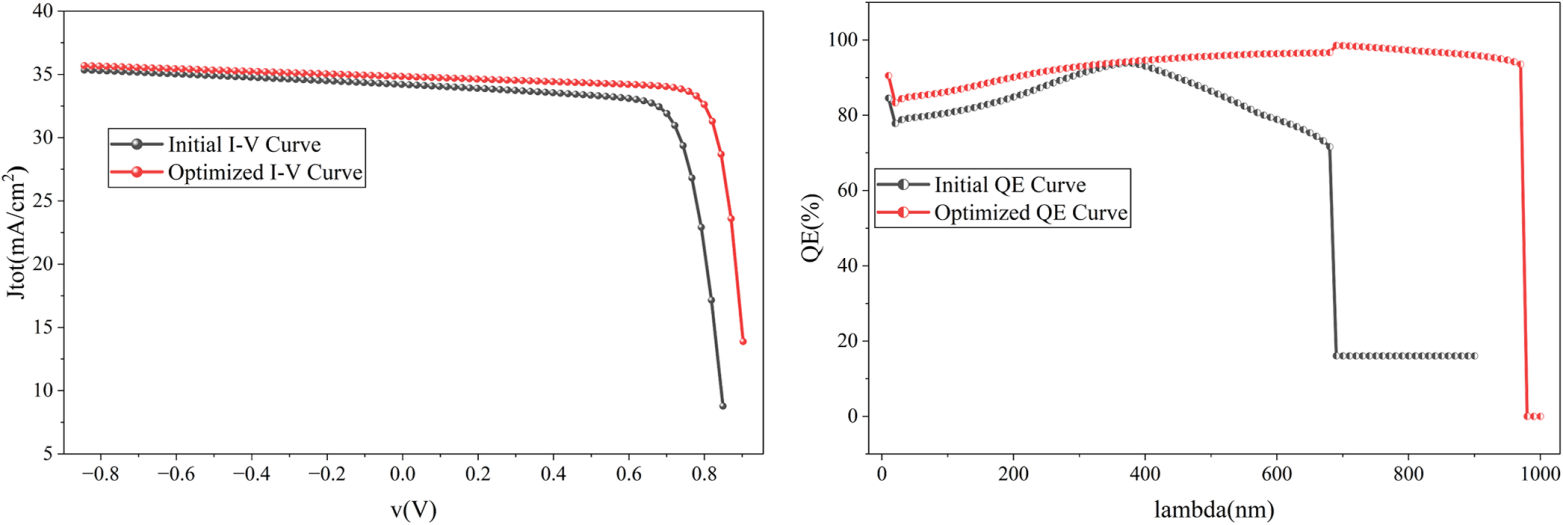
Comparison of the initial and optimized cell’s J–V curve and QE curve.

Figure 21 further clarifies the improvement: the initial energy band diagram had a closer band alignment, which caused higher recombination, whereas the optimized cell has an optimized energy band alignment, which reduces recombination. Additionally, a stronger electric field distribution can also be observed in the optimized cell. These cell conditions significantly improve the overall cell performance.

**Figure 21:**
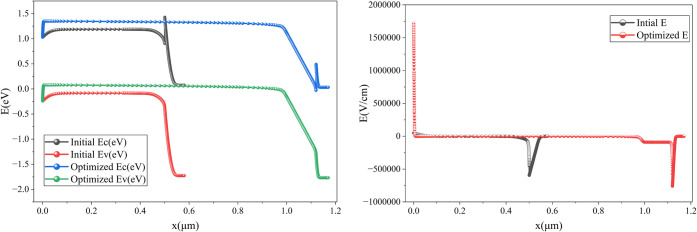
Comparison of the initial and optimized cells’ energy band diagram and electric field distribution.

Figure 22 shows that the overall electron–hole pair generation improved slightly in the optimized cell. Total recombination, on the other hand, dropped significantly, causing optimal output performance.

**Figure 22:**
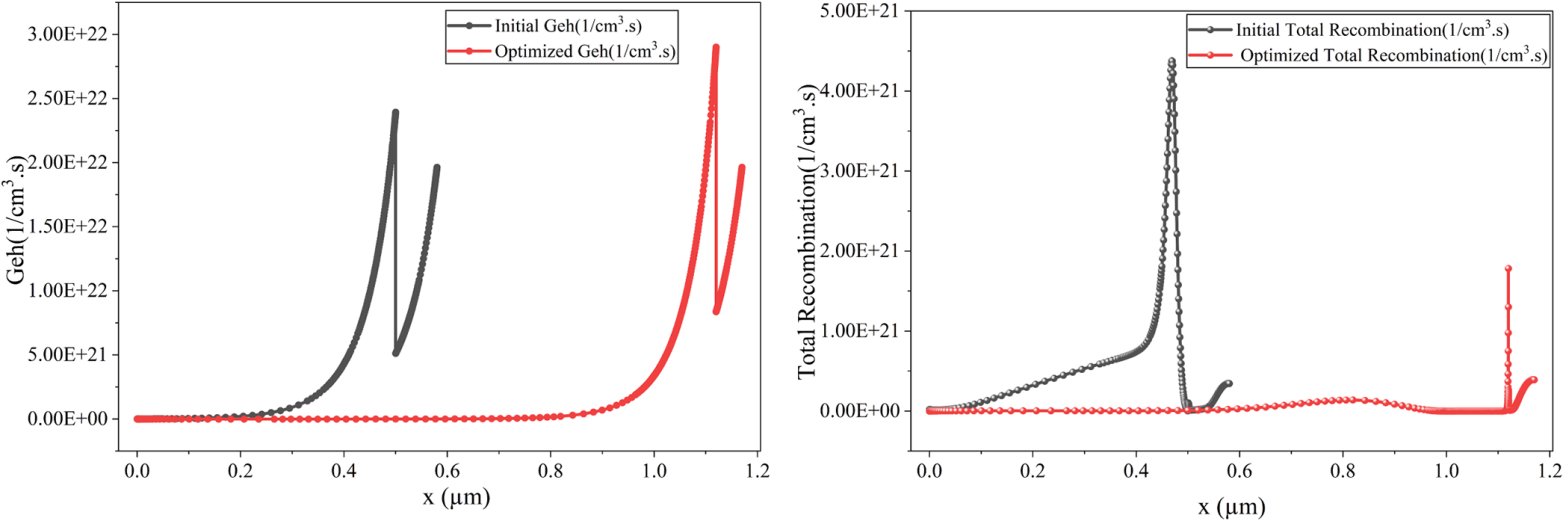
Comparison of the initial and optimized cells’ e–h generation and recombination.

Table 4 clearly highlights the superiority of the proposed cell compared with previous studies, especially when the cost of the cells is considered. Previous cells are either more expensive or deliver lower efficiency. The least expensive cell from previous studies has a cost of 11.92 euros without including the back-contact, which would significantly increase the price if considered. In contrast, the cell from the current study has a combined cost of only 2.232 euros for both the HTL and the back-contact. Therefore, it can be concluded that the proposed cell is not only efficient but also highly cost-effective.

**Table 4:** Comparison between the Previous Study and This Study

Structure	HTM Cost (Euro/g)^a^	Back Contact Cost(Euro/g)	PCE	Comparison
FTO/*n*-TiO_2_/CsSnI_3_/*p*-NiO^11^	13.52 euro	Not mentioned	31.09%	Back contact is ambiguous, Highly efficient with moderate cost
TiO_2_/CsSnI_3_/Spiro-OMeTAD^17^	532 euro	Not mentioned	20.2%	Very expensive compared to PCE
SnO_2_/CsSnI_3_/Spiro-OMeTAD^43^	532 euro	Not mentioned	19.92%	Very expensive compared to PCE
TiO_2_/CsSnI_3_/PTAA^43^	956 euro	Not mentioned	19.89%	Very expensive compared to PCE
SnO_2_/CsSnI_3_/Cu_2_O^43^	11.92 euro	Not mentioned	18.31%	Very expensive compared to PCE
ITO/PCBM/CsSnI_3_/CuI/Au^25^	18.84 euro	344.4 euro	10.10%	Very expensive compared to PCE
WS_2_/CsSnI_3_/Ni(This study)	No Expense	2.232 euro	26.08%	Cost-effective while maintaining high PCE

a Price of materials is taken from Sigma-Aldrich.^37^

Although the cell seems to be a better alternative for low-cost and nontoxic perovskite solar cells, in realistic fabrication, multiple process-level constraints can limit both the reproducibility and scalability of the proposed HTL-free CsSnI_3_/WS_2_ solar cell. The ultralow defect density (∼10^14^ cm^–3^) and precisely graded doping profiles assumed in simulation are difficult to achieve consistently due to the intrinsic instability of CsSnI_3_, particularly the oxidation of Sn^2+^ to Sn^4+^ during film formation and ambient exposure. Variations in deposition techniques (e.g., spin-coating, vapor deposition) can lead to nonuniform film morphology, grain boundary defects, and thickness fluctuations, all of which introduce trap states and increase recombination. Furthermore, achieving a defect-free and well-aligned interface between CsSnI_3_ and WS_2_ is nontrivial, as interfacial strain, lattice mismatch, and chemical incompatibilities can degrade charge transport and device uniformity. When transitioning to large-area fabrication, these issues are amplified, with additional challenges in maintaining homogeneity, controlling stoichiometry, and ensuring batch-to-batch consistency. As a result, the experimentally realized performance is often lower and more variable than simulated predictions, underscoring the importance of robust process optimization, interface passivation, and scalable deposition strategies for reliable commercialization.

## 4 Conclusion

This study examines the design and optimization of HTL-free CsSnI_3_-based perovskite solar cells by using WS_2_ as the electron transport layer (ETL). The optimization process reveals that removing costly hole transport layers (HTLs) and utilizing affordable back-contact materials, such as Ni, can significantly lower production costs without compromising efficiency. The optimized cell achieves a power conversion efficiency (PCE) of 26.08%, an open-circuit voltage (V_oc_) of 0.94 V, a short-circuit current density (J_sc_ *)* of 34.84 mA/cm^2^, and a fill factor (FF) of 79.89%. These results align with the Shockley–Queisser limit, confirming the feasibility of the proposed structure.

The study demonstrates several key findings. Eliminating the HTL not only reduces costs but also improves stability, addressing significant challenges in perovskite solar cells. The optimized CsSnI_3_ absorber (1 μm) and WS_2_ layer thickness are optimized to 0.05 μm, achieving a PCE of 23.86%, V_oc_ of 0.896 V, J_sc_ of 35.65 mA/cm^2^, and FF of 74.68%. Further improvements are achieved by grading the doping concentration of the absorber from 1 × 10^17^ cm^–3^ to 5 × 10^19^ cm^–3^, with a subsequent grading to 1 × 10^16^ cm^–3^, which increases the PCE to 27.27%. A 5 × 10^18^ cm^–3^ doped ETL can further improve the PCE to 29.39%. Additionally, maintaining a defect density of the absorber layer at 1 × 10^14^ cm^–3^ ensures stable performance, with a PCE of 29.39%, V_oc_ of 1.03 V, J_sc_ of 35.28 mA/cm^2^, and FF of 80.84%.

Resistance parameters also play a crucial role in performance optimization. The initial series resistance and shunt resistance of 1.25 Ω and 1000 Ω, respectively, are properly configured for the cell to achieve a 29.39% PCE. The introduction of realistic recombination and interface layers reduces the efficiency to 24.66%. However, adding a thin intrinsic layer improves the cell performance to 26.08%. For the back contact, nickel is chosen due to its cost-effectiveness (2.232 euros/g) while maintaining high performance, making it a viable alternative to traditionally expensive materials.

While the findings are promising, further research is necessary to ensure the long-term stability of the HTL-free configuration under various environmental conditions, including temperature, humidity, and UV exposure. Encapsulation techniques could help mitigate degradation and extend the cell’s lifespan. Scaling the optimized design for commercial applications is another critical challenge. Future studies could focus on large-area fabrication techniques, such as roll-to-roll printing, to assess the viability of mass production. Additionally, interface engineering may further reduce recombination losses by incorporating self-assembled monolayers or passivating interlayers between the absorber and back contact.

Alternative approaches could provide broader comparisons and applications, such as exploring other lead-free perovskite materials with enhanced stability or using TiO_2_ or SnO_2_ as electron transport layers. Cost-effective back contact materials, such as Cu or carbon-based electrodes, might also be viable alternatives. Incorporating self-healing materials or additives in the absorber layer could further enhance the cell’s durability and resilience to defects over time.

This research lays a strong foundation for the development of cost-effective and high-efficiency HTL-free perovskite solar cells. By addressing these challenges and exploring alternative strategies, we can advance toward scalable, stable, and commercially viable solar energy solutions.

## Data Availability

The parameter data used to support the findings of this study is included in the article.
